# Wanting to attend isn’t just wanting to quit: why some disadvantaged smokers regularly attend smoking cessation behavioural therapy while others do not: a qualitative study

**DOI:** 10.1186/1471-2458-14-695

**Published:** 2014-07-07

**Authors:** Fiona E Benson, Karien Stronks, Marc C Willemsen, Nina MM Bogaerts, Vera Nierkens

**Affiliations:** 1 Department Public Health, Academic Medical Centre, University of Amsterdam, Meibergdreef 9, Amsterdam, 1105 AZ, The Netherlands; 2 Department Health Promotion, CAPHRI School for Public Health and Primary Care, Maastricht University, Minderbroedersberg 4-6, 6211 LK, Maastricht, The Netherlands; 3 Present address: VUMC, Department Public and Occupational Health, EMGO+Institute, Van Der Boechorststraat 7, 1081 BT, Amsterdam, The Netherlands; 4 Present address: LUMC, Albinusdreef 2, 2333 ZA, Leiden, The Netherlands

**Keywords:** Smoking cessation, Group therapy, Telephone counselling, Attendance, Low socioeconomic status

## Abstract

**Background:**

Attendance of a behavioural support programme facilitates smoking cessation. Disadvantaged smokers have been shown to attend less than their more affluent peers. We need to gain in-depth insight into underlying reasons for differing attendance behaviour in disadvantaged smokers, to better address this issue. This study aims to explore the underlying motivations, barriers and social support of smokers exhibiting different patterns of attendance at a free smoking cessation behavioural support programme in a disadvantaged neighbourhood of The Netherlands.

**Methods:**

In 29 smokers undertaking smoking cessation group therapy or telephone counselling in a disadvantaged neighbourhood, qualitative interviews were completed, coded and analysed. Major themes were motivations, barriers to attend and social support. Motivations and social support were analysed with reference to the self-determination theory.

**Results:**

Two distinct patterns of attendance emerged: those who missed up to two sessions (“frequent attenders”), and those who missed more than two sessions (“infrequent attenders”). The groups differed in their motivations to attend, barriers to attendance, and in the level of social support they received. In comparison with the infrequent attenders, frequent attenders more often had intrinsic motivation to attend (e.g. enjoyed attending), and named more self-determined extrinsic motivations to attend, such as commitment to attendance and wanting to quit. Most of those mentioning intrinsic motivation did not mention a desire to quit as a motivation for attendance. No organizational barriers to attendance were mentioned by frequent attenders, such as misunderstandings around details of appointments. Frequent attenders experienced more social support within and outside the course.

**Conclusion:**

Motivation to attend behavioural support, as distinct from motivation to quit smoking, is an important factor in attendance of smoking cessation courses in disadvantaged areas. Some focus on increasing motivation to attend may help to prevent participants missing sessions.

## Background

Smoking is a major preventable cause of death in Europe [1]. It is of particular importance for those of low socioeconomic status (SES), as they are more likely to smoke [2-4] and have been shown to be less likely to succeed in quit attempts [5,6]. For these reasons, it is important to provide evidence-based measures to disadvantaged smokers to maximise every quit attempt’s chance of success. One such measure is the combination of behavioural support, in the form of a group therapy or telephone counselling, and pharmacotherapy.

Several studies show positive effects of behavioural support although the effect size differs [7,8]. The effect size is dependent, amongst other things, on attendance rate. A Cochrane review on the effect of behavioural support in combination with pharmacotherapy [9] found, on pooling 38 studies most of which offered 4 or more sessions, that there was a small but statistically significant positive effect of increased attendance of the behavioural therapy, be it face-to-face or by phone, on long-term quitting behaviour.

Those of low SES, in particular, have been shown to exhibit poor attendance of smoking cessation behavioural support, attending less often than their higher SES counterparts [10,11]. Hiscock et al. [10] suggested that addressing this problem of low attendance rates would increase success rates in disadvantaged smokers.

The existing research into attendance in community-based smoking cessation programmes gives insight into the background characteristics of participants and typically concentrates on whether quit attempts are successful, rather than participants’ attendance behaviour. Moreover, as far as they address attendance, these are mainly quantitative studies. For example, studies among Latino smokers [12,13] found that completion of the intervention or assessment was more likely when participants were less depressed, saw less ‘pro’s’ of smoking, were unemployed [12], and had less initial confidence in their ability to quit, better health in relation to peers, in addition to higher income [13]. Two qualitative studies have been identified which discuss smoking cessation and attendance [14,15]. The first, Lee et al. [14], is a study which looks at the reasons smokers and health care professionals give for smokers’ lack of cessation. This study, however, only looks at participants who have ceased treatment (and remained smoking). Allan et al. [15] is also a qualitative study of participants reasons for failing to complete a smoking cessation intervention, however, this intervention gives participants incentives to quit. The focus was on the effect of the incentives and, again, only participants who had missed sessions were included. None of the existing papers looks at attendance behaviour with reference to a theory of motivation, however, such a theory is necessary to deepen an understanding of motivations to attend behavioural support programmes.

Self-determination theory (SDT) [16], is a theory of motivation which has been used to consider the role of motivation in behavioural persistence in an educational setting [17] and in exercise adherence in the longer term [18]. There are three types of motivation which are part of a continuum from non-self-determined to self-determined [19] (see Table 1). The first type of motivation is termed “amotivation”, or non-self-determined behaviour. The second and most common type of motivation, is “extrinsic motivation”, or motivation to achieve a particular goal. And the third type of motivation is “intrinsic motivation”, which is doing something for the joy of the activity itself and is fully self-determined. Within extrinsic motivation there are four different levels of motivation, moving from less self-determined to more self-determined. “External regulation” is a behaviour performed only to satisfy an external demand or reward and is the least self-determined or autonomous. Slightly more self-determined are “Introjected regulations”, which are behaviours performed to prevent guilt or anxiety or for ego reasons, such as pride. People are motivated in order to maintain feelings of self-worth. It involves internalising some regulation, but not accepting it fully as one’s own. The next most self-determined is “Identified regulation”, in which a behavioural goal is consciously valued, so that the action is accepted or owned as personally important. And finally, the most self-determined of the extrinsic motivations, is “Integrated regulation”, which occurs when an external goal is fully internalised and assimilated with an individual’s other values and needs. This is closest to intrinsic motivation, however it is still done to satisfy an external goal. The more self-determined a behaviour is the more likely it is to be undertaken by an individual.

**Table 1:** The self-determination continuum

**Type of motivation**	**Amotivation**	**Extrinsic motivation**				**Intrinsic motivation**
**Type of regulation**	Non-regulation	External	Introjected	Identified	Integrated	Intrinsic
**Type of behaviour**	Non-self-determined					Self-determined

Adapted from Deci & Ryan [20].

Making a behaviour more self-determined can be achieved by fulfilling one or more of the three basic needs for relatedness, autonomy and competence [20]. Autonomy is the ability to guide one’s own behaviour [21]. Relatedness is feeling “securely connected to and esteemed by others and to belong to a larger social whole” [21], p.251, and competence is the skills which enable meaningful actions to be taken.

This theory also discusses support, which is important in attendance. Social support, from those in an individual’s environment or a counsellor, can help individuals to fulfil the three basic needs. Autonomy support helps as it involves people important to the individual creating a social environment which features “eliciting and acknowledging perspectives, supporting self-initiative, offering choice, providing relevant information and minimizing pressure and control” [22], p.731. Autonomy support in turn enhances feelings of competence toward a behaviour and thus an individual’s intentions toward that behaviour [22]. Relatedness, which can also be provided by autonomy supportive relationships, helps by encouraging individuals to undertake a behaviour because it is “valued by significant others to whom they [feel] (or would like to) feel connected” [19], p.64.

Based on this theory it seems important to study underlying basic needs in relation to attendance behaviour, and to begin this work with a qualitative study to inform future quantitative studies. The aim of this study is to understand in-depth the motivations of low SES smokers who attend many, as well as, fewer sessions of smoking cessation behavioural support, regardless of smoking status. Our research question is: Do low SES smokers who regularly attend smoking cessation therapy differ in their motivations, barriers and social support, from those who do not?

## Methods

### Setting

The study took place in The Hague, which is one of the four largest cities in The Netherlands, with a population of 506,366 [23]. People living in a disadvantaged, multi-ethnic neighbourhood were offered free smoking cessation behavioural support with or without pharmacotherapy. The behavioural support consisted of either group therapy or telephone counselling (see Table 2). Participants should choose their support type. Group therapy [24] consisted of 9 sessions of group training +/- two sessions of extra activities (17/29 participants). Telephone counselling [25] consisted of 7 standard sessions (12/29 participants). Participants began the behavioural support while still smoking, but were expected to stop at a certain time during the course. Both behavioural support types were available in Dutch and Turkish.

**Table 2:** Characteristics of both behavioural support formats offered

**Characteristic**	**Group therapy**	**Telephone counselling**
**Number of sessions**	9 standard weekly sessions, sometimes supplemented by +/- 2 extra sessions, in this disadvantaged area, on open topics chosen by the group, such as stress management.	7 standard sessions over the course of 3–4 months, with up to 5 extra sessions if the participant has relapsed or has not stopped after the first session.
**Number of participants**	8 – 12	1
**Session duration**	1.5 – 2 hours	Session 1: 30 minutes
		Subsequent sessions: 15 minutes.
**Timing of official stop date**	Session 4	Between Sessions 1 and 2.
**Languages available**	Dutch and Turkish	Dutch and Turkish
**Gender of group participants**	Offered in mixed groups (in Dutch) or all male and all female groups (in Dutch & Turkish).	N/A
**Permitted to keep participating if not stopped on official stop date**	Yes	Yes
**Content of sessions**	The course increases motivation and teaches self-control techniques using learning methods such as group discussion, working in pairs, role play, individual and group exercises, visualisation, and homework exercises.	1. The participant’s motivation is increased using motivational interviewing and they are prepared for the first few days after stopping.
	Topics include:	2. Withdrawal symptoms.
	- Self observation,	3. Desire to smoke.
	- Analysis of tempting situations,	4. tempting situations.
	- Decreasing nicotine use,	5. Topic of choice (including stress, weight gain, gloom, boredom or loneliness.)
	- Motivation,	6. Prevention of relapse.
	- Behavioural rules,	7. Follow – up (after 3 months).
	- Suddenly stopping,	Sessions 2 – 7 all include some time spent on maintaining motivation. [25]
	- Changing smoking behaviour,	
	- Rewarding yourself,	
	- Coping with desire to smoke,	
	- Cognitive restructuring,	
	- Coping with social pressure,	
	- Relapse prevention after a slip. [24]	

### Study design

This study is part of a larger quantitative study of the effectiveness of a smoking cessation program in a disadvantaged, multi-ethnic neighbourhood in The Hague. In this study, people who enrolled to attend smoking cessation behavioural therapy offered in a disadvantaged area of The Hague were asked to participate and were then followed-up on four occasions (a baseline interview, an interview at 4 weeks, 6 months and 1 year after their agreed quit date). In this qualitative study, in-depth interviews were used because we aimed to understand the process individuals went through with regard to attendance, as well as their behaviour and perceptions.

### Recruitment

Participants were recruited between October 2011 and May 2012 by purposive sampling from participants in the larger study. They were selected for their variety in attendance behaviour, from complete attendance through to attending only the intake session, and for their diversity of ethnic backgrounds, reflecting the disadvantaged neighbourhood’s population.

Recruitment for the study was done by two of the authors and two additional interviewers (see acknowledgements), who contacted participants by phone to ask if they were willing to participate. Contact was attempted five times on different days and at different times. Of the 72 participants enrolled in the quantitative study at that time, 47(65%) were asked to participate and 29(62%) took part. Recruitment was stopped when a purposive sample (i.e. gender, age, ethnicity, marital status, attendance and smoking behaviour) and data saturation were obtained. Of the 18(38%) who did not participate, 12(26%) refused due to lack of time or no interest in the subject of stopping smoking or both, 3(6%) couldn’t be contacted, 2(4%) refused due to health reasons, and 1(2%) refused due to having only done an intake interview and not the full course. Of those 18 (2 unknown), the attendance status was: present at all (3(17%)), missed >2 (8(44%)), and Intake only or dropped out prior intake (5(28%)).

### Participant characteristics

The characteristics of participants can be viewed in Table 3. The majority of participants used pharmacotherapy, including varenicline, bupropion or nicotine replacement patches/lozenges.

**Table 3:** Participant characteristics

**Characteristics**	**Number of participants n(%)**
**Gender (n(%)):**	
**Male**	17(59)
**Female**	12(41)
**Nationality (n(%)):**	
**Dutch**	9(31)
**Turkish**	15(52)
**Surinamese**	4(14)
**Antillean**	1(3)
**Marital status (n(%)):**	
**Married**	15(52)
**Partner**	1(3)
**Single (unmarried/no partner)**	7(23)
**Divorced**	6(21)
**Therapy type (n(%)):**	
**Group therapy**	17(59)
**Telephone counselling**	12(41)
**Pharmacotherapy (n(%)):**	
**Yes**	22(76)
**No**	7(24)
**Ages (years):**	
**Range**	24 - 71
**Mean(SD)**	46.10(12.25)
**Self-reported smoking status after course (n(%)):**	
**Stopped**	9(31)
**Smoked less**	13(42)
**Unchanged**	7(23)
**Fagerstrøm Test of Nicotine dependence score (n(%)):**	
**Very low dependence (0–2)**	5(17)
**Low dependence (3–4)**	6(21)
**Medium dependence (5)**	6(21)
**High dependence (6–7)**	8(28)
**Very high dependence (8–10)**	4(14)
**Mean score(SD)**	5(2.31)
**Participants’ estimate of the chance of success of this quit attempt measured prior to beginning behavioural support (0–10):**	
**Mean(SD)**	7.75(2.05)
**Participants’ motivation to permanently stop during the programme measured prior to beginning behavioural support (0–10):**	
**Mean(SD)**	8.70(1.35)

### Data collection

The data was collected during face-to-face (28) and telephone (1) semi-structured interviews done in the months following the behavioural support. Most face-to-face interviews were conducted in the participant’s home or, on occasion, in a public place (e.g. library). Participants received a €10 honorarium for participation. Informed consent for participation in the larger study had already been obtained from all participants. Under Dutch law medical ethical approval was not required for this study. Participants were given a participant number to ensure their anonymity and identifiable information was kept confidential.

A topic list based on themes from the literature, such as expectations of the course, social support received by participants, and depressed mood, was developed as a guide for the interviews. Open questions were asked such as: “Some participants missed sessions now and again. How did you come to attended all the sessions?”, “Did you ever consider not going?” “Could you tell us about the times you considered not going?”, or “Could you tell us about the times that you didn’t attend a session?” Interviews of between 30–90 minutes were recorded and interviewers made field notes after the interviews, describing where the interviews had taken place, and indicating any occurrence which may have altered the information given in the interview, such as the presence of a family member. Interviews were transcribed verbatim into either Turkish or Dutch. All Turkish transcripts were then translated verbatim from Turkish into Dutch. Transcripts were not returned to participants for comment or correction due to the expected high proportion of participants unable to read or write to a sufficient level to undertake this task, though verifiable facts were checked with the quantitative data for veracity.

Four female interviewers undertook the interviews, all of whom had undertaken tertiary studies in Health Sciences or anthropology. Two interviewers were experienced qualitative interviewers and two had had training prior to undertaking interviews. The interviewers were not involved in executing the intervention. No researchers in this project had any competing interests.

### Analysis

The data was analysed using an inductive approach [26,27] which allows researchers to develop a framework of the underlying processes existing in the data [27]. Two researchers (FB) (NB), independently developed codes and refined these into a single code hierarchy based on the analysis of 6 initial interviews. Disagreements were brought to the attention of a third researcher (VN) for discussion and decision. A further 4 interviews were then coded by two interviewers (FB) (NB) at intervals throughout the coding process to ensure congruence. Extra themes emerging from the data were added to the coding hierarchy on the basis of these additional interviews and, subsequently, as they occurred in the remainder of the interviews. Coding was applied by researchers in the software package MAXQDA (Version 10), which aided in data retrieval. Analysis identified themes occurring in the data and thematic charts were developed which aided in synthesis and summarisation of the data and in looking for all potential associations in the data. During the initial analysis we found a pattern within the motivations to attend that fitted the self-determination theory [19]. Subsequently motivations to attend were assigned along the self-determination continuum [20] by a single researcher (FB or NB) and this was checked by a second researcher (VN). Social support was also analysed with regard to the conditions which foster self-determined behaviour [20]. Barriers were analysed with regard to the source of the barrier, and these were subsequently cross-referenced with the motivations of the individuals involved. Differences of opinions were discussed and resolved prior to finalisation.

Major themes were interview guided. These were motivations to attend the course, social support both outside and within the course, and barriers to attendance. Through thematic charting minor themes emerging from the data were identified. We present here the strongest patterns in the data which help to clarify the research question. The results are given in sections for each of the major themes, and these are then subdivided by minor themes.

We also looked for the influence of other factors on attendance such as reasons to do the course, ethnicity, experiencing a stressful life event, depression, medication, gender, and expectations of the course.

## Results

During the analysis we saw that the participants fell into one of two main groups with regard to their motivation to attend, barriers to attendance and social support. These groups were those who missed up to 2 of the scheduled sessions of group therapy or telephone counselling (frequent attenders) and those who missed more than 2 of the scheduled sessions of group therapy or telephone counselling, including those who had an intake interview only (infrequent attenders). In the case of telephone counselling, a session was not considered missed if it was re-scheduled and then took place.

Findings in the results section will be given with reference to these two groups. The groups were not evenly distributed; There were 21 frequent attenders and 8 infrequent attenders, and more infrequent attenders were male. The participant characteristics of the two groups were otherwise similar (see Table 4).

**Table 4:** Participant characteristics of frequent and infrequent attenders

**Characteristics**	**Frequent attenders**	**Infrequent attenders**
**Gender (n(%)):**		
**Male**	10(48)	7(88)
**Female**	11(52)	1(13)
**Nationality (n(%)):**		
**Dutch**	6(29)	3(38)
**Turkish**	11(52)	4(50)
**Surinamese**	4(19)	-
**Antillean**	-	1(13)
**Marital status (n(%)):**		
**Married**	10(48)	5(63)
**Partner**	1(4.8)	-
**Single (unmarried/no partner)**	5(24)	2(25)
**Divorced**	5(24)	1(13)
**Therapy type (n(%)):**		
**Group therapy**	12(57)	5(63)
**Telephone counselling**	9(43)	3(38)
**Pharmacotherapy (n(%)):**		
**Yes**	17(81)	5(63)
**No**	4(19)	3(38)
**Ages (years):**		
**Range**	24 – 66	26 – 71
**Mean(SD)**	45.19(11.39)	48.50(14.86)
**Self-reported smoking status after course (n(%)):**		
**Stopped**	8(38)	1(13)
**Smoked less**	4(19)	3(38)
**Unchanged**	9(43)	4(50)
**Fagerstrøm Test of Nicotine dependence score (n(%)):**		
**Very low dependence (0–2)**	2(10)	3(38)
**Low dependence (3–4)**	5(24)	1(13)
**Moderate dependence (5)**	4(19)	2(25)
**High dependence (6–7)**	7(33)	1(13)
**Very high dependence (8–10)**	3(14.3)	1(13)
**Mean score(SD)**	5.43(1.99)	3.88(2.85)
**Participants’ estimate of the chance of success of this quit attempt measured prior to beginning behavioural support (0–10):**		
**Mean(SD)**	8(1.69)	7.13(2.80)
**Participants’ motivation to permanently stop during the programme measured prior to beginning behavioural support (0–10):**		
**Mean(SD)**	8.84(1.12)	8.38(1.85)

### Motivations to attend the course

#### ***Enjoyment of the course***

A third of the frequent attenders mentioned enjoyment of the sessions, the atmosphere and the group, as a reason for attendance. This percentage differed between the two types of courses: half of the group therapy attendees mentioned enjoyment while only one participants undertaking telephone counselling did

*Interviewer (I): And there were moments when I thought, we-e-ll..* [that you didn’t want to go?]*Respondent (R): No, I always enjoyed it. I enjoyed going.*(1016, male, 59, Surinamese, group therapy, stopped smoking, frequent attender)

This was in contrast with the infrequent attenders, where only one participant mentioned enjoyment of the course. These participants often named no longer enjoying the sessions after the initial few sessions. This seemed to be related to perceived barriers which will be described later. According to self-determination theory intrinsic motivation is a strong motivator to continue undertaking a behaviour. It would appear that this is the case for attendance behaviour in this group. A minority of people who mentioned enjoyment of the course mentioned their desire to stop as a motivation for attendance, thus intrinsic motivation to attend often seems to be unrelated to their motivation to quit.

#### ***Wanting to stop smoking***

Smokers’ desire to stop was mentioned by over a third of frequent attenders as a motivation for attendance. They mentioned their desire to stop smoking and alluded to the fact that this decision and taking action toward it was in their own hands, as illustrated by the below quote. The desire to stop was mentioned by a single infrequent attender.

*Because I made my own decisions. I went for it. I went for it. I said, “I’m going to stop.” And I did stop.*(1039, male, 49, Turkish, group therapy, smokes less after relapse, frequent attender)

The desire to stop and the view that this was in their own hands can be interpreted through the self-determination theory as an extrinsic motivation and one of a more self-determined nature (integrated), because participants have internalised a goal which is not inherently enjoyable, making it their own aim, and they subsequently feel autonomous in their pursuit of this goal.

Interestingly, only a minority of the frequent attenders who reported this desire to stop as a motivator for attendance were intrinsically motivated. Thus it would seem that this desire is a powerful motivator for attendance even in those who don’t report enjoying sessions.

#### ***Commitment to attendance***

Frequent attenders often indicated that they chose to attend of their own free will, for example, they organized their schedule around the course, they placed a high value on appointments or they would not have considered cancelling. No infrequent attenders mentioned commitment to attend. One frequent attender alludes to attendance being a ritual for her, and though this was only stated by her, the sense of the ritual of attendance was also present in interviews with other frequent attenders.

*I had told the temping agency, “You mustn’t call me on Friday, because I am doing the course.” I was always there. I could stay at home and then at about 1.30 I could go and sit with my friend who lives on …nearby here. And then, at ten to 3, a quarter to 3, I would leave. It was a ritual for me.*(1023, female, 66, Surinamese, group therapy, stopped smoking, frequent attender)

Another participant stressed the value he and his wife placed on appointments, no matter who they were with.

*R: Well, we set great store by appointments.I: Mmm.R: I mean, whether it’s the G.P. or the dentist…*(1030, male, 55, Turkish, group therapy, smokes less, frequent attender)

Telephone counselling participants did not need to commit to being in a certain place at a certain time each week. They often mentioned having re-scheduled sessions for various reasons including death in the family to being currently occupied by another task. This participant indicates the flexibility involved in session scheduling.

*I did, now and again, yes, yes, I did now and again. I can’t at the moment… You’re phoning at a bad time. Okay, and then mostly she would phone me back in the evening. Sometimes I was doing the shopping or I would specify a time, then she would phone me at that time. Or you could plan another appointment in the evening, a telephone appointment*.

(2029 male, 54, Dutch, telephone counselling, smokes less, infrequent attender)

For this, they needed to have the competence to plan convenient appointments with their counsellor, or to reschedule if required.

*I put the children to bed at such and such time. Just like I did now [*for this qualitative interview*]. For example, I’d say, “On er…Monday, for example, I won’t be at home. So I can’t talk. Tuesday one of the children sleeps at this time. The other doesn’t bother me. You can phone me, at this time, for example.” That’s how it went.*(2009, Female, 33, Turkish, telephone counseling, stopped smoking, frequent attender)

Organisation of their schedule around the course, placing importance on the course and going out of their way for the course can be interpreted as an extrinsic motivation. We interpreted it as a more self-regulated extrinsic motivation because it involves placing importance on an activity which is not inherently enjoyable and then personally committing to ensuring that it is possible to meet the demands of this activity. Autonomy (the personal choice to make the course a priority) and competence (the ability to re-organise one’s personal schedule) are required in order to achieve this. The frequent attenders also indicated their feelings of competence (organising schedule around course) and autonomy (importance of keeping appointments) which are not typically named by Infrequent attenders. Interestingly, only some of those frequent attenders who reported this also reported enjoyment of the course or intrinsic motivation, thus commitment to attendance appears to be a powerful motivation in and of itself. This lack of intrinsic motivation, but commitment to the course, is also seen in the majority of telephone counselling participants.

### Social support received by participants

Social support received by participants undertaking the course came from sources either outside (e.g. partner, family, friends or people in the environment, such as colleagues or acquaintances) or within (e.g. counsellor or other participants) the course.

#### ***Social support outside the course***

Remarkably, both frequent and infrequent attenders faced much negativity toward their quit attempt, or attendance of the course, in the social environment outside the course. And just under half the participants in both groups told no-one or were selective in who they told about their quit attempt. Within this context, however, people also received support, as detailed below.

A minority of participants actually stated that they felt supported in the social environment outside the course. In these cases they perceived support from a certain group or individual (e.g. partner, family, friends). More often participants mentioned receiving support, despite not mentioning that they felt supported. This took many forms including encouragement, as in the quote below, which we saw as relatedness, or advice or personal stories from stoppers who were known to them, or people not purchasing cigarettes for them or not smoking around them, which we saw as autonomy support

*I had a girlfriend who had also stopped and, there she was, and then she said to me every time, “I’m proud of you,” and all that, and that made me feel good.*(2031, female, 48, Dutch, telephone counselling, stopped smoking, frequent attender)

The advice or personal stories allowed them to practice self-appraisal or bench-mark themselves against others, as seen here.

*Well, if my father has stopped smoking, then everyone will stop.*(2026, Male, 34, Turkish, telephone counselling, smokes less, frequent attender)

Almost half of the frequent attenders reported receiving autonomy support or relatedness or both, however, support often came with a lack of support within the same social group. The most common example was receiving support from non-smokers but not from smokers, as in the quote below, though this was not always the case. Infrequent attenders reported receiving little support.

R: Most of the friends who don’t smoke…

I: Mmm

R: “You have done well.”

I: Mmm

R: “Good that you have stopped.” But them who smoke don’t say this (laughs).

I: (Laughs) They won’t be satisfied.

*R: There were satisfied* [previously]*. If they smoke, I can’t smoke, so…*

(2027, female, 31, Turkish, telephone counselling, smokes less, frequent attender)

#### ***Social support within the course***

Support within the course from the counsellor is discussed below. Support from the group was only received by group therapy participants, so while it is not discussed here it is important to note that some group therapy frequent and infrequent attenders mentioned feeling supported and motivated by the group. However, negative aspects of the group were also mentioned by half of the frequent and infrequent attenders.

#### ***Support from the counsellor***

Many frequent and infrequent attenders felt motivated and understood by the counsellor, that the counsellor was happy to have them at sessions and that they were given individual attention or feedback. Half the participants felt that the counsellor did a good job, was enjoyable to speak to, or knew what he or she was talking about, as illustrated below.

*Well, that the, let’s say, the teacher, er, the course manager, he did it really good, really very good, explains everything very well, and he motivated people, and he did an awful lot so that people stop, but the people, they didn’t come.*(1063, male, 67, Antillean, group therapy, smokes less, infrequent attender)

Frequent attenders received more tips, appraisal opportunities or tangible support with organisation of medication from the counsellor than infrequent attenders. This participant describes how his counsellor helped him to get reimbursed pharmacotherapy.

*I had to pay for it myself, but that woman* [the counsellor] *helped me so I didn’t have to pay. She got in touch with the pharmacy, the sickness fund, last year then, and then I didn’t have to pay for it.*(1011, male, 43, Turkish, unchanged, frequent attender)

From the theory we see that the opportunities for appraisal, and tangible help which is perceived as support, can be seen as autonomy support. Frequent attenders reported receiving autonomy support from the counsellor more often than infrequent attenders. Feeling motivated and understood by the counsellor, and the counsellor being happy to have them at sessions and giving individual attention and feedback, can be seen as relatedness. Feelings of relatedness to the counsellor were often mentioned by both frequent and infrequent attenders. For many participants, the counsellor was able to build a good rapport and supply the need for autonomy support or relatedness.

More telephone counselling participants felt relatedness to the counsellor than did group therapy participants. This relatedness came in the form of feeling motivated by the counsellor or receiving compliments from the counsellor.

#### ***Negativity toward the counsellor***

Negative comments about the counsellor were made by some participants, especially amongst those attending group therapy. While frequent attenders seem to be commenting on the counselling style, such as the counsellor not stopping another participant from speaking at length, infrequent attenders commented on unprofessional behaviour, such as this participant describing the counsellor gossiping with participants about absent participants.

*R: Yes, they were always gossiping about others. About other colleagues from the group and in fact the counsellor just got involved and I thought that was a bit, well .. yeah.O: And the counsellor didn’t say something like, “Well, shall we get on with the lesson.”R: No, she just got involved in the discussion, too. Joined in. Well, I just felt that this was, whether it was true or not, I don’t like it.*(1008, male, 41, Turkish, group therapy, unchanged, infrequent attender)

The infrequent attenders who had these experiences with the counsellor also mentioned no longer enjoying the course after this. Thus it is possible that these experiences impact on intrinsic motivation and, as a result, on persistence of attendance behaviour. However, it is also possible that this is a rationalisation for no longer being motivated to attend..

### Barriers to attendance

A third major theme identified was barriers to attendance. During the analysis, three types of barriers emerged: external, internal and organisational barriers.

#### ***External barriers***

The majority of reasons for non-attendance mentioned by frequent attenders were external reasons which were specific to the individual, such as illness, going on holiday or having to work. External reasons were also given by infrequent attenders. Those reasons were mainly given by those attending group therapy. This could be because group therapy participants were unable to re-schedule sessions.

Some frequent attenders also reported going out of their way to attend the course and overcome barriers to participate in sessions. They mentioned taking part in sessions despite illness or tiredness from work. Thus barriers which may have been a reason for non-attendance or an excuse were overcome by frequent attenders, but not by infrequent attenders. Only one of these participants who reported going out of the way for the course stated their commitment to attendance (see section on Motivation). However, more self-determined motivations were seen in four of the six participants who mentioned overcoming barriers, where four reported wanting to stop and two of these were also intrinsically motivated to attend. No infrequent attenders mentioned going out of their way to attend sessions.

#### ***Internal barriers***

Only one frequent attender gave an internal reason for non-attendance, which was feeling guilty due to having relapsed, as illustrated below.

*I: You didn’t attend the last session, and was it also…R: If you ask me, it was also due a bit to my, er, failure, simply, that I actually had to admit that I hadn’t quite succeededI: YesR: That in fact, I, er, yes, on purpose, by accident, I forgot.I: Yes.R: That is what I shall say. The last appointment.I: OkR: YesI: Yes.R: Because, in fact, the only thing I could say was that that I really had not succeeded. And that upsets me an awful lot. (laughs)*(2008, female, 50, Dutch, telephone counselling, unchanged, frequent attender)

It is possible that this participant did not want to tell her counsellor because she wanted to avoid confrontation or feeling uncomfortable.

In contrast to the frequent attenders, many infrequent attenders mentioned internal barriers. More than half the infrequent attenders mentioned no longer finding it enjoyable or useful to attend, as this participant indicates.

*First few times it was nice* [before many participants dropped out](1063, male, 67, Antillean, smokes less, infrequent attender)

Reasons given for this loss of enjoyment included non-attendance by others (previously known to the participant or not), something occurring during sessions, or quitting and thus not finding the sessions useful any more.

We see that some infrequent attenders noted a loss of enjoyment, or intrinsic motivation, as a barrier to attendance, in comparison to the third of frequent attenders who mentioned intrinsic motivation as a motivator of behaviour seen previously. These people may have begun with intrinsic motivation to attend, but seemed to lose it during the course because of what happened during the sessions.

#### ***Organisational barriers***

While no frequent attenders mentioned experiencing organisational barriers to attendance, the majority of infrequent attenders gave an organizational reason for non-attendance of at least one, if not more, of their sessions. The reasons given included that they had turned up at the agreed place and time and no-one was there, that they were not contacted about starting a course, that sessions had been cancelled, or that they had been put into an inappropriate form of training (group therapy instead of telephone counselling), as seen in the quote below.

*I attended 2–3 times. I said at the time that if I am driving instructor, in fact, yes, had little time to come to the meetings and that I preferred to have it with him by telephone.*(1014, male, 45, Turkish, group therapy, smokes less, infrequent attender)

Some infrequent attenders made attempts to overcome the organizational barriers, however, others did not.

Organisational barriers to attendance were not reported by frequent attenders, while almost all infrequent attenders experienced them. The near universality of this experience in Infrequent attenders suggests that these barriers may occur regularly, however, it is important to note that none of these participants experienced more self-determined motivations to attend, thus it is possible that they may have been excuses not to attend.

### Other themes

Other factors which may have had an impact on attendance were also looked at, such as reasons to do the course, ethnicity, experiencing a stressful life event, depression, use of smoking cessation pharmacotherapy, gender, and expectations of the course. The only one of these which had an impact was gender, where we found that most women were frequent attenders. There were no additional differences to those already seen in the division between frequent and infrequent attenders.

## Discussion

### Findings in this study placed in the context of the literature

Smokers who frequently attend smoking cessation behavioural support offered in a disadvantaged neighbourhood were found to have more self-determined motivations to attend. Moreover, they had more external, rather than internal or organisational barriers to attend. Frequent attenders also experience more autonomy support and relatedness than infrequent attenders.

The main finding with regard to motivation to attend in this disadvantaged group was that frequent attenders were more likely to exhibit intrinsic motivation or more self-determined extrinsic motivations to attend, compared to infrequent attenders, despite the fact that smoking cessation is not an inherently enjoyable activity [28]. This has not been reported before in the literature. Similar to Lee et al. [14], we found the desire to quit to be a factor in attendance, however, intrinsic motivation seemed to be an equally important factor in attendance and these two factors were not typically mentioned together, so this factor seems to be as important as a desire to quit. Similar to one US study on intervention participation in low SES, ethnic minority attendees of a behavioural intervention [29], we identified internal and external barriers to attendance, however, we additionally found that frequent attenders experienced no organizational barriers to attendance, while infrequent attenders almost universally experienced such barriers.

With regard to social support, most participants, regardless of attendance status, experienced much negativity about their quit attempt or their course participation in the social environment outside the course. In this context, frequent attenders, as compared with infrequent attenders, experienced more autonomy support and relatedness from the counsellor, similar to the abovementioned study [29], and also from people outside the course similar to another US study examining the role of social influence on attendance in a behavioural intervention [30], which. Our findings suggest that a supportive environment inside and outside the course can positively influence attendance behaviour.

Also, those who did group therapy exhibited more intrinsic motivation than those who did telephone counselling. It is possible that the flexibility of the sessions somehow compensated this lack of intrinsic motivation, so that many telephone counselling participants were also frequent attenders, despite undertaking what might be a less enjoyable form of counselling. To our knowledge this has not been previously mentioned in the literature.

### Implications

Besides the suggestions given above, the results imply that more focus on keeping participants motivated to attend may improve attendance in people from low socioeconomic groups. Intrinsic motivation seems to be an important motivator of continued attendance. This suggests that interventionists and counsellors should make courses enjoyable to ensure maximum attendance. For example, in group therapy, regularly using techniques to increase relatedness, such as ice-breakers and re-energizers [31], could be considered. This also increases social support within the course, which may also positively influence attendance. For other participants who may not enjoy sessions, it may be possible to increase their attendance by flexible scheduling, such as through rolling groups which have been successful in disadvantaged smokers in the UK [32]. It is also possible that increasing social support outside the course, possibly through involvement of family members or friends [27], may increase attendance.

Interestingly, the relationship between attendance and smoking cessation in our study is not clear-cut and further study needs to be done to elaborate on this point.

### Strengths and limitations

To our knowledge this is the only European qualitative study of a multi-ethnic group in which disadvantaged smokers give underlying reasons for their patterns of attendance at non-incentive based smoking cessation behavioural therapy programmes, focussing mainly on those who frequently attend, whose perspectives have not been shown before.

However, some limitations need to be considered before drawing conclusions. Firstly, it is possible that a national change in subsidy allocation to pharmacotherapy during the study period biased the results. There was much confusion about how an individual’s pharmacotherapy would be paid for by insurance companies. We expected this to be a cause of poor attendance, but did not find it to be so. It may have increased the number of frequent attenders because of a feeling of needing to attend all sessions to gain the reimbursement, however, we do not feel the results were biased because only a minority of frequent attenders mentioned this issue as a motivation for attendance, despite the participants being specifically asked about it.

Secondly, in this research it was not always possible to clearly separate cause and effect. For example, most infrequent attenders mentioned organisational barriers. While removing organisational barriers to attendance through improved and more regular communication with participants both prior to and during the course may lead to increased attendance, we noted that none of the participants who experienced organisational barriers experienced more self-determined motivations to attend, so it is possible this kind of barrier could be an excuse in infrequent attenders. Participants who didn’t overcome their barriers to attend may experience cognitive dissonance [33], and it could be that organisational barriers are rationalisations which help reduce dissonance and mask a loss of motivation. This suggests that investment in increasing motivation is perhaps the most important focus in increasing attendance.

Amongst those who refused to participate in the research, the majority were infrequent attenders, while these were the minority group in our research. It is possible that this may have biased our findings, however, we don’t think this is the case because recruitment was continued until data saturation was reached.

## Conclusion

Attendance behaviour is an understudied aspect of multi-session smoking cessation behavioural support, especially among smokers in disadvantaged areas. The motivations, barriers and social support of those who attend frequently are different from those who attend less frequently. If free behavioural support is offered, participants with varying levels of motivation to attend will enrol. Strategies to increase intrinsic motivation to attend counselling sessions should be an integral aspect of multi-session smoking cessation behavioural support, in addition to strategies to increase motivation to quit.

## Competing interests

The authors declare that they have no competing interests.

## Authors’ contributions

FB participated in study design, data acquisition, analysis and interpretation of data, and drafting the paper. KS contributed to data interpretation and drafting.MW contributed to data interpretation and drafting. NB contributed to data acquisition, analysis and interpretation. VN conceived of the study, participated in its design, contributed to data interpretation and drafting of the manuscript. All authors received and approved the final manuscript.

## Pre-publication history

The pre-publication history for this paper can be accessed here:

http://www.biomedcentral.com/1471-2458/14/695/prepub
